# A review on the role of fatty acids in colorectal cancer progression

**DOI:** 10.3389/fphar.2022.1032806

**Published:** 2022-12-12

**Authors:** Malvina Hoxha, Bruno Zappacosta

**Affiliations:** Department for Chemical-Toxicological and Pharmacological Evaluation of Drugs, Faculty of Pharmacy, Catholic University Our Lady of Good Counsel, Tirana, Albania

**Keywords:** cancer, colorectal cancer, fatty acid, PUFA, eicosanoid

## Abstract

Colorectal cancer (CRC) is the third leading cause of mortality in cancer patients. The role of fatty acids (FA) and their metabolism in cancer, particularly in CRC raises a growing interest. In particular, dysregulation of synthesis, desaturation, elongation, and mitochondrial oxidation of fatty acids are involved. Here we review the current evidence on the link between cancer, in particular CRC, and fatty acids metabolism, not only to provide insight on its pathogenesis, but also on the development of novel biomarkers and innovative pharmacological therapies that are based on FAs dependency of cancer cells.

## 1 Introduction

In the early 1950s, Medes et al., described the role of glucose carbon and acetate in the fatty acid synthesis in neoplastic tissues (Medes et al., 1953). This study was the first to determine that lipogenesis occur in neoplastic tissues. Subsequently, numerous studies confirmed the importance of fatty acid biosynthesis for cancer cell growth and survival. The regulation of lipid synthesis, the respective metabolism, as well as their uptake and degradation are essential for the maintenance of the cellular physiology; hence the perturbation of these processes can impact the cancer development (Snaebjornsson et al., 2020). During the last 15 years, the rewiring of cellular metabolism in cancer cells has been widely discussed (Hanahan and Weinberg, 2011; La Vecchia and Sebastian, 2020).

Colorectal cancer (CRC) is the third most common malignant neoplasm, with a high rate of mortality, and incidence, which is predicted to increase by 60% in 2030 (Ferlay et al., 2013). Wnt (Thompson 2014), KRas (Pupo et al., 2019) and p53 (Labuschagne et al., 2018) are among the CRC drivers and regulators of cancer metabolism.

## 2 The fatty acids (FAs) overview: From biochemistry to metabolism

Fatty acids (FAs) are carboxylic acids that are involved in energy storage and are absorbed from food, or synthesized *de novo*, endogenously from acetyl CoA, which involve the FAs uptake and esterification into cell membranes as phospholipids (Ratnayake and Galli, 2009; Currie et al., 2013). Esterified FAs govern some of cell membrane physical properties, or can be released through phospholipase action. Besides, exogenous FAs can be stored as triglycerides and cholesterol esters (Ferreri et al., 2016).

Saturated fatty acids (SFA) contain no double bond. Other FAs that contain carbon double bonds with an even number of carbon atoms are termed unsaturated fatty acids. Monounsaturated fatty acids (MUFAs; i.e. palmitoleic acid, 16:1) are FAs that contain one double bond, while polyunsaturated fatty acids (PUFAs, i.e. arachidonic acid, 20:4) contain more than one double bond.

### 2.1 *De novo* biosynthesis

**FIGURE 1:**
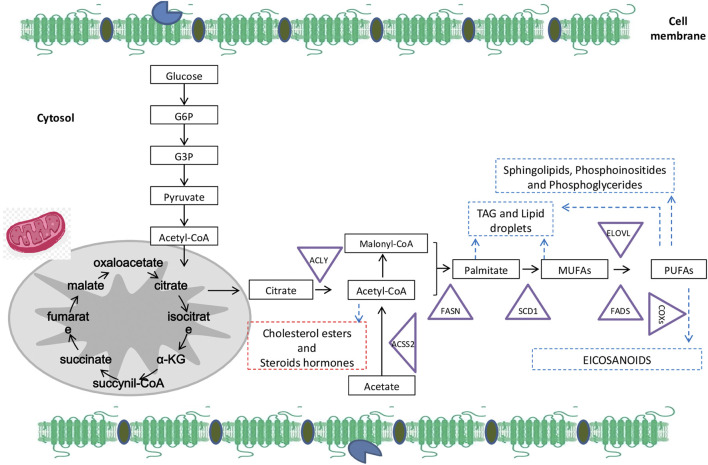
Overall fatty acids (FAs) biosynthesis and metabolism: The *de novo* synthesis starts from glucose that generates citrate. Citrate can be cleaved by ATP-citrate lyase (ACLY) to acetyl-CoA that is also metabolized from acetate generated by acetyl-CoA carboxylases (ACSS2). After carboxylation, acetyl-CoA (as well as malonyl-CoA) is condensed by FA synthase (FASN) to give rise to palmitate. Palmitate is catalyzed by the stearoyl-CoA desaturase (SCD1) to synthetize monounsaturated fatty acids (MUFAs) such as oleate and palmitoleate by introducing a double bond between carbons 9 and 10 of the acyl-CoA substrate. MUFAs are desaturated by a desaturase in plants to generate polyunsaturated fatty acids (PUFAs). Moreover, cyclooxygenases (COXs) are able to metabolize PUFAs from 20-carbons to synthetize bioactive lipids with pro or anti-tumor activity (according to the substrate). Whereas, exogenous or endogenous PUFAs are elongated and desaturated by FA elongases (ELOVLs) that add two carbons and desaturases (FADSs) -that add a double-bound between carbon. Abbreviations: α-KG, α-ketoglutarate; G3P, glyceraldehyde-3- phosphate; G6P, glucose-6-phosphate.

Tumor cells are characterized by both a high FAs supply, and upregulation of lipogenesis (Tiwary et al., 2019; Koundouros and Poulogiannis, 2020). As shown in Figure 1, *de novo* synthesis of FAs starts from acetyl-CoA originated from carbohydrate or protein metabolism, as well as from FA oxidation.

Acetyl-CoA is catalyzed through FA synthase (FASN) in 16:0 PA (palmitic acid), which is further elongated in 18:0 SA (stearic acid). Δ9-Desaturase is responsible of the transformation of 16:0 PA into 16:1 (palmitoleic acid), and 18:0 into 18:1 (oleic acid) (Ratnayake and Galli, 2009). Endogenous monounsaturated fatty acids (MUFA) are synthesized by stearoyl-CoA desaturases (SCD) and Δ9-desaturases from saturated fatty acids that are rate-limiting for MUFA synthesis (Green et al., 2010).

### 2.2 Polyunsaturated and essential fatty acids

Besides SFA and MUFAs biosynthesis, different families of PUFAs exist, such as omega-3 (ω-3) PUFA, omega-6 (ω-6) PUFAs, omega-7 (ω-7) PUFA, omega-9 (ω-9) PUFA (Das, 2004). Eicosapentaenoic acid (EPA), docosapentaenoic acid (DPA), α-linolenic acid (ALA), and stearidonic acid (SDA) belong to the ω-3 PUFAs (Shahidi and Ambigaipalan, 2018). Linoleic acid (LA) is part of the ω-6 PUFAs. Palmitic acid and oleic acid belong to ω-7, and ω-9 PUFAs respectively.

**FIGURE 2:**
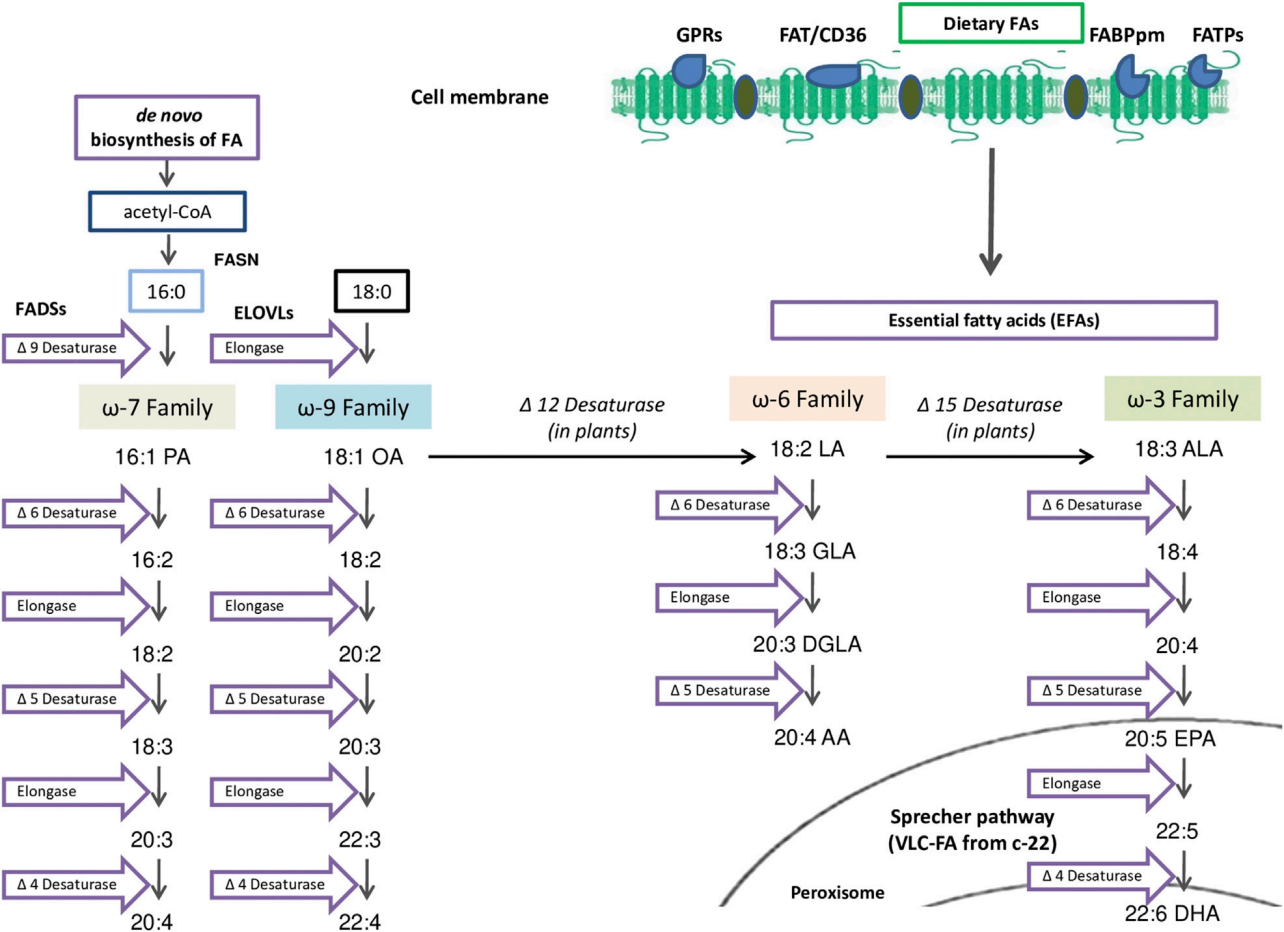
Polyunsaturated FA (PUFAs) and Essential FA (EFAs) metabolism: PUFAs can be provided by exogenous uptake from the surrounding microenvironment facilitated by specialized transporters such as the fatty acid transport protein (FATPs), the fatty acid–binding protein-plasma membrane (FABPpm), G-protein coupled receptors (GPR) and fatty acid translocase lipid microdomains (FAT/CD36). In mammals, essential FAs (EFAs) such as Linoleic Acid (LA 18:2 ω-6) or α-Linolenic Acid (ALA 18:3 ω-3) are both uptaken because they cannot be synthetize endogenously. LA and ALA metabolized competitively by ELOVLS and FADSs that can introduce double bonds at the Δ5 or Δ6 position to synthetize long-chain FAs (LC-FA) or subsequently, very-long-chain FAs (VLC-FA) in peroxisome by Sprecher pathway.

ω-7 and the ω-9 PUFAs that derive from palmitoleic acid (PA 16:1 ω-7) and oleic acid (OA 18:1 ω-9) respectively, are synthesized in mammals, unlike essential fatty acids (EFAs), ω-3 PUFAs (linolenic acid (ALA 18:3, ω-3)), and ω-6 PUFAs (linoleic acid (LA 18:2 ω-6)) that cannot be synthesized in humans (Ratnayake and Galli, 2009). The enzymes responsible of elongation and desaturation reactions of EFAs (ω-6 and ω-3 PUFAs) are located in the endoplasmic reticulum (Grammatikos et al., 1994). Δ6-Desaturase is responsible of the elongation of stearidonic acid (SDA, 18:4) in eicosatetraenoic acid (ETA 20:4 ω-3) (Figure 2), which is further desaturated by Δ5-desaturase giving rise to EPA 20:5. In regard to the ω-6 family, arachidonic acid (AA 20:4) is produced from LA (18:2 ω-6), which is further desaturated to gamma-linolenic acid (GLA 18:3). In addition, because of the sharing of the same enzymes, the transformation of LA to AA (ω-6 family), is competitive with that of ALA to EPA (ω-3 family) (Grammatikos et al., 1994). 22:5 ω-6 and 22:6 ω-3 (docosahexaenoic acid, DHA) are produced from AA and EPA, following elongation and Δ4-desaturation respectively. Sprecher et al. reported that the biosynthesis of DHA occurs through two sequential elongations of AA and EPA, which is followed by Δ6-desaturation, and ß-oxidation in the peroxisome (Sprecher et al., 1995). Interestingly, this pathway, which implies a significant role of peroxisomes in the biosynthesis of the very long-chain PUFAs, has been proposed as an unconventional pathway (Qiu 2003).

Since Δ5 and Δ6-desaturases are slow in humans (Δ5>Δ6), the supplementation of EPA and DHA to bypass Δ5 and Δ6-desaturases is extensively recommended in subjects with an ω-3 PUFAs deficient Western Diet. In addition, in humans the DHA is low following supplementation with ALA or EPA, hence DHA supplementation is pivotal (Richard and Calder, 2016).

### 2.3 FA uptake

In studies using membrane models, it has been reported that uncharged FAs enter into cells by passive diffusion (flip-flop) (Simard et al., 2008). Overwhelming evidence showed that in mammalian cells, the FAs uptake occurs by a wide variety of integral or membrane associated proteins. It has been recognized that most of the exogenous FAs are rapidly distributed into tissues through particular mechanisms. For this reason, FA uptake can be regulated according to the metabolic needs in order to prevent the potential effects of excessive FA accumulation (Ehehalt et al., 2006).

Different proteins are involved in mammalian cells FA uptake: FATP (fatty acid transport protein), FABPpm (fatty acid–binding protein-plasma membrane), GPR (G protein coupled receptors), and lipid microdomains FAT/CD36 (fatty acid translocase).

The FATPs (fatty acid transport proteins) (FATP1–6) family are a multifunctional set of proteins present in plasma membrane, or intracellular organelles, that are implicated through different functions in lipid metabolism. FATP1, FATP2 and FATP4 are involved in either fatty acid transport, or very long chain fatty acid activation (Black et al., 2009).

Besides FATPs, also the plasma membrane associated FA-binding proteins (FABPs), known as intracellular lipid chaperons are small cytosolic proteins that bind reversibly to long chain fatty acids (C16-C20), and eicosanoids (Mishkin et al., 1972; Furuhashi and Hotamisligil, 2008). Nine (FABP1–FABP9) out of 12 FABPs forms discovered are expressed in humans (Storch and Thumser, 2010; Smathers and Petersen, 2011). FABPs are implicated in FAs storage, import, export, and signaling (Smathers and Petersen, 2011). Once inside, FAs are bound by cytosolic-FABP, which stimulates not only FA absorption, but also its cytoplasmic redistribution (McArthur et al., 1999). For instance, the intestinal isoform of FABP can bind to free long chain fatty acids and lead to fatty acid absorption (Furuhashi and Hotamisligil, 2008; Smathers and Petersen, 2011). FABP genes expression in human CRC was studied. Prayugo et al. reported that numerous FABP genes are altered in CRC patients, and that FABP6 could be a potential biomarker of colorectal adenocarcinomas (Prayugo et al., 2021). Bile acids that are increased in colon adenomas and cause DNA damage and apoptosis in colonic epithelium are thought to be responsible of the raised FABP6 expression in early-stage carcinogenesis in CRC (Imray et al., 1992; Fujii et al., 1993). Interestingly, FABP6 expression is also thought to be linked to insulin-like growth factor (IGF) signaling in CRC (Nowakowska-Zajdel et al., 2011). FABP4 levels are also increased in CRC patients (Zhang et al., 2019).

Over the past two decades, G protein coupled receptors (GPRs) have been systematically “de-orphanized”. Regarding the fatty acid uptake, specifically GPR40, 41 and 43 are capable of recognizing either short (GPR41 and GPR43), medium or long chain FA (GPR40), responsible of mediating the signalling regulatory effects of these nutrients. GPR120, highly expressed in the intestine, regulates FA-induced glucagon-like peptide one secretion (Husted et al., 2017). In addition GPRs are also involved in CRC. FFAR (free fatty acid receptor), specifically FFAR2 (GPR43), FFAR3 (GPR41), that are activated by short chain fatty acids produced in the colon are involved in colon tumorigenesis (Wu et al., 2012; Lavoie et al., 2020). Controversial studies report either the role of FFAR2 in potentiating colon tumorigenesis, or suppressing it through activation of caspases (Hatanaka et al., 2010; Tang et al., 2011). FFAR3 can stimulate cell proliferation, and enhance colon tumorigenesis (Wu et al., 2012). GPR109A highly expressed in colon is involved in colon cancer (Kimura et al., 2020). G protein-coupled estrogen receptor (GPER) that is implicated in FASN modulation can suppress CRC progression, despite some controversial studies reporting a promoting role of GPER in CRC (Santolla et al., 2012 .; Gilligan et al., 2017).

Lipid rafts are microdomains present at the plasma membrane of cells that act as mediators between the internal and external compartments of the cell (Ikonen 2001). The fatty acids inside the lipid raft tend to form a solid compartment. Fatty acid translocase (FAT/CD36) association with lipid rafts can regulate FA uptake. Although CD36 is overexpressed in different cells such as: macrophages, endothelial cells, its role in fatty acid binding and uptake is not well known (Jay and Hamilton, 2018). It is reported that lipid rafts are involved in metastasis, cell migration/survival, and signal transduction in different type of cancers (Ma 2007; Patra 2008), specifically in colorectal cancer (Rakheja et al., 2005; Chapkin et al., 2007). Orlandi and Fishman (Orlandi & Fishman, 1998) were the first to report the implication of lipid rafts in CRC. Lipid rafts are involved in cell proliferation, cell adhesion and death in CRC (Jahn et al., 2011). Insulin-like growth factor-I (IGF-I) receptor segregation regulate pro and anti apoptotic effects in colon adenocarcinoma cell lines (Remacle-Bonnet et al., 2005). Fatty acid synthase (FASE) that is responsible of fatty acids synthesis is overexpressed in CRC, and its inhibition has a substantial role in lipid raft synthesis (Jahn et al., 2011).

To sum-up, in mammals, FAs can be synthesized endogenously or uptaken from diet through passive diffusion, or by the regulation of specific sets of membrane proteins that ensure FAs uptake. Moreover, even if plants are able to synthesize ω-3 or ω-6 PUFAs, in mammals they should only be provided from diet, and both ω-3 and ω-6 PUFAs families are essential for homeostasis.

## 3 Functions of fatty acids in cancer cells

The alteration of FA synthesis is responsible of different aspects of cancer growth, such as the energy storage, cell proliferation and survival. Besides lipids, also PUFA derivatives, the eicosanoids (from C20 fatty acids) play a role in tumorigenesis and tumor microenvironment (Röhrig and Schulze, 2016).

### 3.1 Membrane structure and fluidity

Physiologically, cells membranes are mainly composed of phospholipids. PUFAs are esterified in the sn-2 position, while MUFAs in the sn-1 position are esterified in phospholipids, maintaining the structural integrity of the lipid bilayer membrane, and conferring fluidity. FAs can be unesterified thanks to the activity of the phospholipase released in the cytosol as free-fatty acids (Swinnen et al., 2019). The alteration of enzymes involved in lipid metabolism is responsible of the very significant change of membrane lipid composition in cancer cells in respect to normal cells. The higher degree of membrane saturation makes the cells less sensitive to oxidative stress induced by chemotherapeutic agents, protecting cancer cells from oxidative stress-induced cell death hampering with chemotherapy and redox homeostasis (Rysman et al., 2010). In addition, the higher degree of saturated fatty acid and cholesterol confers rigidity to the cell membrane, interfering with growth, and anti-growth factors, followed by the transduction of the signals to the inside of the cell. The altered growth factor signaling enhances cell growth, and uncontrolled division.

The growth factor receptors signaling are either affected by the content of membrane lipids, or explicit mutations/or amplification in genes encoding growth factor receptors (Arkhipov et al., 2013). Yes-associated protein (YAP) expression is increased in CRC, and YAP oncogene expression is stimulated by Wnt/β-catenin signaling. Higher levels of YAP and β-catenin are found in the tumor cell nuclei (Tanton et al., 2022). Ouahoud et al. reported that the risk of developing CRC is reduced by statins, thus cholesterol plays an important role in CRC through Wnt/(YAP) growth signaling pathway (Ouahoud et al., 2022; Tanton et al., 2022).

### 3.2 Substrate for ATP synthesis

Mitochondrial oxidative phosphorylation (OXPHOS), and glycolysis are the sources of ATP in normal cells, in contrast to cancer cells that obtain ATP mostly from glycolysis (Shiratori et al., 2019). The energy metabolism of cancer cells is a potential therapeutic target. β-oxidation, or fatty acid oxidation (FAO) produce energy through FA degradation. In cancer cells there is a high request for ATP, for phosphorylation reactions, and DNA/RNA replication (Ma et al., 2018). FAO inhibition is associated with induction of apoptosis, cytotoxic accumulation of long-chain fatty acids, potentially correlated with the dysregulation of reactive oxygen species (ROS) production, mitochondrial damages, and NADPH homeostasis (Menendez and Lupu, 2007; Leamy et al., 2013).

### 3.3 Energy storage

Diacylglycerol is esterified into triacylglycerol, and brings to lipid droplets (LD) biogenesis. LDs are storage oragnelles that play an important role in signaling, energy metabolism and in the production of inflammatory mediators (Zoncu et al., 2011; Jaishy and Abel, 2016; Olzmann and Carvalho, 2019). LDs are accumulated in a variety of cancer cells and their catabolism is tightly coupled to energetic metabolism. Moreover, LDs catabolism has been also linked to cell signaling and cancer cell proliferation, resistance to death, and aggressiveness.

The *de novo* lipid synthesis and remodeling, the increased lipid uptake, the cross-talk through tumors and other cells are some of the functions of LDs in tumor cells (Cruz et al., 2020). Interestingly, in CRC, the LDs are major sites for prostaglandin E_2_ (PGE_2_) synthesis, an important immune suppressive eicosanoid involved in tumor cell proliferation (Accioly et al., 2008). PGE_2_ is responsible of inhibiting apoptosis, enhancing angiogenesis, and cell proliferation. Data have shown that either LD, or LD-derived PGE_2_ can enhance the proliferation of epithelial cells (Cruz et al., 2020). In addition, in CRC, mPGES and COX-2 are also localized in LDs. In conclusion, FASN inhibitors reduced the LD and PGE_2_ synthesis in IEC-6 H-rasV12 cells (Accioly et al., 2008).

### 3.4 Pro-tumorigenic signalling molecules

Phosphatidylinositol 3-kinase (PI3K)/Akt pathway is the most common dysregulated signalling pathway in human cancers, which could be activated by growth-factor receptor tyrosine kinases (RTKs), and is also associated with the activation of rapamycin (mTOR) complexes. In addition, PI3K–AKT signaling inhibits β-oxidation and lipolysis, and promotes lipid synthesis (Fazolini et al., 2015). Anabolic metabolism and *de novo* lipogenesis are activated through mTORC1 mechanisms. Cholesterol and lipids may activate the mTOR signalling pathway (Sabatini 2006). mTORC1, and mTORC2 are stabilized by phosphatidic acid, which is synthesized *de novo* by fatty acid metabolism (Menon et al., 2017). Data have shown that mTORC1 complex is also stimulated by exogenous unsaturated fatty acids, such as exogenous oleic acid through the *de novo* synthesis of phosphatidic acid (Fingar and Blenis, 2004; Menon et al., 2017). Liu et al., showed that PI3K plays a role as inducer of chemoresistance in CRC (Liu et al., 2017), and in CRC patients, PIK3CA mutation leads to the activation of PI3K/Akt signaling, and chemotherapy resistance (Wang et al., 2018a).

Phosphatase and tensin homolog (PTEN), a negative regulator of PI3K signaling, is also a tumor suppressor with an important role in the metabolism of glucose and lipids, that works in the nucleus. SREBP and FASN induction brings to increased *de novo* lipogenesis, as a consequence of hepatic loss of PTEN (Stiles et al., 2004; Chen et al., 2018). PTEN changes in CRC are linked with BRAF mutations, lymph node metastases (Salvatore et al., 2019). Wang et al., showed that SIRT3/PTEN/AKT/RHEB/MTOR/HIF1α signal pathway promotes tumor proliferation (Wang et al., 2018b).

Data have reported that around 40% of colon cancers have a KRAS mutation, which are associated with a higher tumor aggressiveness (Malumbres and Barbacid, 2003). The signal transduction of KRAS effector proteins reduce apoptosis, and increase angiogenesis. The mutant KRAS upregulates the fatty acid synthase, ATP citrate lyase, and acetyl coenzyme A carboxylase, controlling *de novo* lipogenesis (Pupo et al., 2019). Plasma membrane proteolipid composition is altered by the presence of EPA and DHA through the suppression of KRAS phenotypes both *in vivo* and *in vitro*, and by the alteration of pERK (Fuentes et al., 2018). Interestingly, KRAS gene mutation is associated with a reduced response to anti-epidermal growth factor receptor (EGFR) agents (Lievre et al., 2006; Phipps et al., 2013). Point substitutions in codons 12 and 13, the most common KRAS mutations are considered as negative predictors of EGFR antibodies response. This can bring to the development of new personalized therapies, based on the type of KRAS mutation (Dinu et al., 2014).

Vascular endothelial growth factor receptor (VEGFR), platelet-derived growth factor receptor (PDGFR), fibroblast growth receptor (FGR) are receptor tyrosine kinases (RTKs) cell surface receptors characterized by an extracellular domain (ectodomains), whose activation induces PI3K/AKT/mTOR, and RAS kinase pathways (Jin et al., 2019). In CRC different mutations can activate RTKs. The mutation of epidermal growth factor receptor (EGFR) leads to C16 saturated fatty acid production (Bollu et al., 2015).

### 3.5 Eicosanoids remodel the tumor microenvironment

PUFAs are the precursors of prostanoids involved in inflammation. Specifically, the mediators deriving from C20-FAs are named eicosanoids. Prostanoids that include leukotrienes, thromboxanes and prostaglandins are potent signaling molecules synthesized by a diverse set of enzymes during inflammation. Among them, the PGE_2_ is the major cyclooxygenase (COX) product, that has a significant role in cardiovascular, gastrointestinal, and renal system, and is also involved in cancer. Several studies, in fact indicate higher levels of PGE_2_ in colon, colon, lung and neck cancer (Lenihan-Geels et al., 2016). Inflammatory cells and fibroblasts infiltrate within the tumor microenvironment (TME) in the intestine interacting through chemokines and cytokines with CRC cells, to enhance tumor progression and growth. PGE_2_ inhibition might suppress CRC progression (Aoki and Narumiya, 2017).

## 4 Potential therapeutic strategies in CRC: New trend and future prospective

**FIGURE 3:**
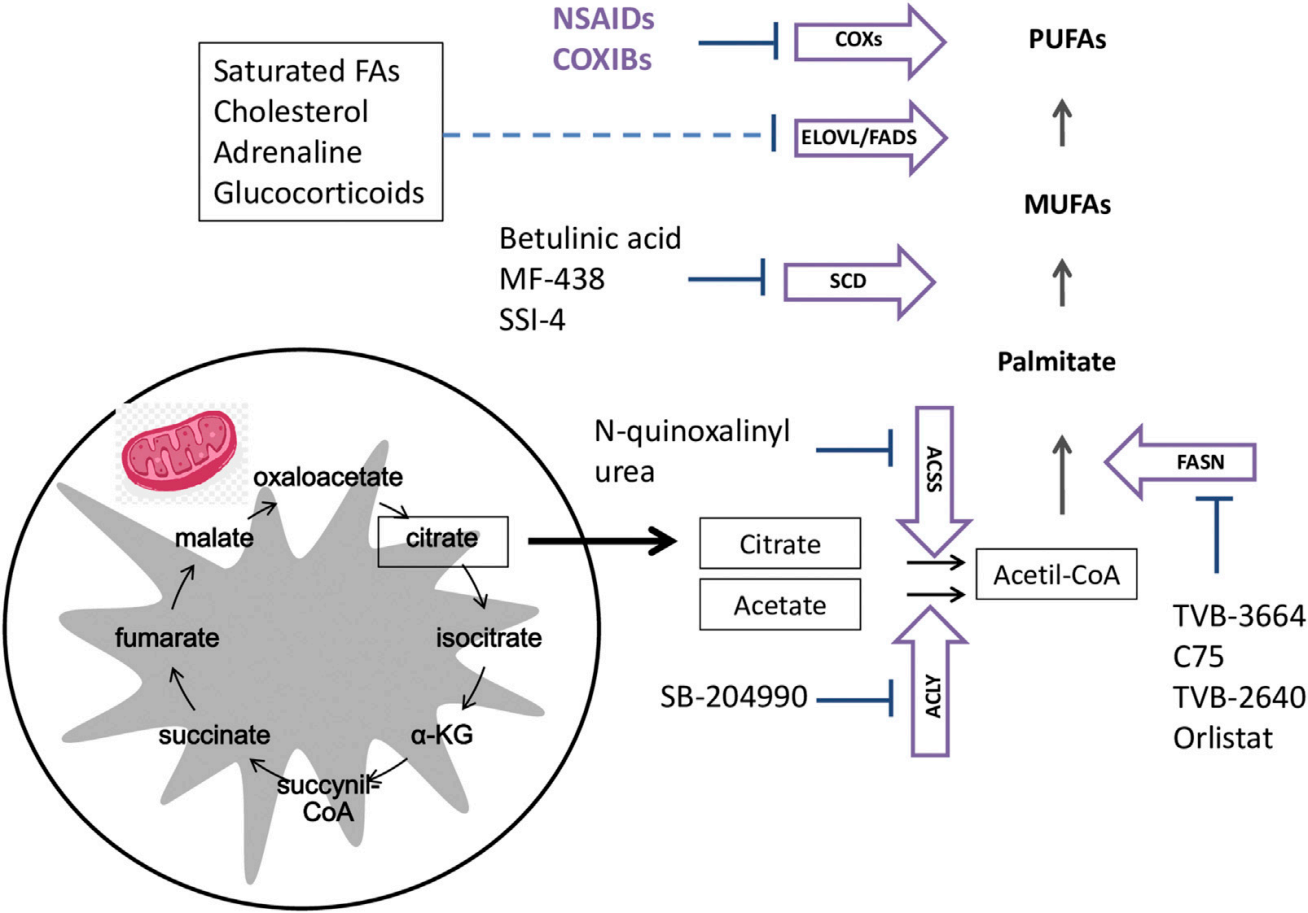
Targeting enzymes involved in fatty acid oxidation or bioactive lipid synthesis: Cancer cells obtain fatty acids (FAs) from *de novo* lipogenesis and exogenous uptake. FAs and their synthetic products can be subsequently stored as lipid droplets or used for acetyl-CoA production through β-oxidation. Palmitate is the main product of *de novo lipogenesis* and it is generated from citrate or acetate through the enzymatic activities of ACSS or ACLY, respectively to give rise to acetyl-CoA. Acetil-CoA can be metabolized by FASN to Palmitate (16:0) that will be metabolized by SCD1 to MUFA and, subsequently desaturated and elongated through the activity of ELOVLs and FADs to produce a diverse group of PUFAs. Finally, PUFAs are substrate of COXs enzymes that oxygenate these FAs to bioactive lipids with a differential activity in tumor initiation and progression. The enzymes involved in FA oxidation, as well as those able to give rise to bioactive lipids can be a therapeutic target to hamper tumor progression. Abbreviations: COXs: cyclooxygenases; ELOVLs: FA elongases; FADSs: FA desaturases; ACLY: ATP-citrate lyase; ACSS2: acetyl-CoA carboxylases. FASN: FA synthase; SCD1: stearoyl-CoA desaturase; MUFA: monounsaturated fatty acids; PUFAs: polyunsaturated fatty acids; α-KG, α-ketoglutarate.

Considering the implication of FAs in cancer pathogenesis, new pharmacological therapies can be developed by targeting FA metabolic reprogramming. As shown in Figure 3, targeting the enzymes involved in fatty acid oxidation (FAO), or bioactive lipids from PUFAs, as well as designing a personalized food-plan as coadjuvant therapeutical strategy might likely hamper tumor progression.

### 4.1 Harboring the anabolic metabolism and oncogenic signaling

#### 4.1.1 Targeting the fatty acid synthase

FASN is the enzyme responsible of the conversion of acetyl-CoA and malonyl-CoA in palmitate. FASN is a precursor of cholesterol and glycerophospholipids and has a significant role for energy storage. Studies have demonstrated that *de novo* lipogenesis supplies cancer cells with sufficient energy, and signaling lipid molecules to enhance fast cell proliferation (Buckley et al., 2017). Two FASN isoforms (FASN I and FASN II) have been denoted among living organisms. FASNI is present in fungi and animals, whereas, FASNII in prokaryotes and plants (Röhrig and Schulze, 2016). The rationale for using FASN inhibitors in combination with chemotherapy derives from the well known cellular effects of FASN inhibition such as: the palmitate synthesis blockade, alteration of plasma membrane structure, oncogenic signal transduction inhibition (e.g. β-catenin, Wnt, and Akt signalling), gene expression reprogramming, and tumor cell apoptosis induction (Mullen and Yet, 2015). The inhibition of FASN causes a reduction in *de novo* palmitate synthesis and consequent palmitoylation, an alternative approach to block oncoprotein activation. Examples of palmitoylated proteins include tubulin and EGFR, as well as RAS-family GTPases that require palmitoylation to promote tumor formation activity. For these reasons, the existing drugs that target FASN are one advantageous aspect of this approach (Ko and Dixon, 2018). In the last years, different FASN inhibitors have been studied. Cerulenin (an antifungal antibiotic capable of inhibiting the FASN reductase activity), as well as Orlistat (a drug widely used for obesity treatment), C75, TVB-3166 and TVB-2640 were used in preclinical models of breast cancer. In particular, C75 was showed to prevent breast cancer development in HER2-transgenic mice. In addition, C75 blocked DNA replication and triggered apoptosis (Menendez and Lupu, 2017). In preclinical models of mesothelioma (Gabrielson et al., 2001), as well as in renal (Horiguchi et al., 2008), lung (Relat et al., 2012), and prostate cancer (Zadra et al., 2019) it has been shown how FASN inhibition was able to modulate the proliferation and to induce apoptosis in cancer cells. Regarding CRC, only one inhibitor was tested successfully. In particular, TVB-3664 that was used in CRC patient-derived xenografts (PDXs), reduced tumor volume in 30% of the cases, with no significant toxicity. The antitumor effect of TVB-3664 was associated with variations in lipid composition. Akt, AMPK, Erk1/2 were among the oncogenic pathways altered (Zaytseva et al., 2018). In view of its roles in enhancing anabolic metabolism, and oncogenic signaling, FASN has been widely studied for its therapeutical role. However, the *de novo* lipid synthesis should be studied in CRC not only in the Apc-model. In addition, Yekaterina et al., showed that another FASN inhibitor, TVB-2640 (currently on Phase I and II clinical trials) studied in patients with solid tumor showed a good tolerability profile (Yekaterina et al., 2016; National Cancer Institute, 2018). TVB-3693, and TVB-3664 were also studied in CRC cells. Findings report that the FASN inhibition response can be measured through the basal levels of pAMPK and pAkt, and FASN inhibitors can have a potential effect either in the treatment of early CRC, or as prevention therapy (Cruz et al., 2014; Yekaterina et al., 2016; Drury et al., 2022). Indeed, low mitochondrial respiration, low glycolysis, are a result of FASN deletion, suggesting that fatty acid synthesis upregulation foster tumorigenesis (Bueno et al., 2019; Drury et al., 2022).

### 4.2 Limiting the metabolic substrates for lipogenesis

#### 4.2.1 Targeting the ATP citrate lyase

Citrate and coenzyme A (CoA) are converted to acetyl-CoA and oxaloacetate through ATP citrate lyase (ACLY), an enzyme linking lipid and glycolitic metabolism. Low levels of fatty acids*,* and high levels of carbohydrates activate the *de novo* synthesis of fatty acids in cancer cells, which is followed by the conversion of glucose to pyruvate by glycolysis. In Krebs cycle, pyruvate is converted through pyruvate dehydrogenase in acetyl-CoA. The rest of the puyrvate is excreted as lactate (Granchi 2018). The regulation of cholesterol or fatty acids synthesis in obese or hyperlipidemic patients, could be potentially achieved through the use of ACLY inhibitors. The interest of shifting towards the use of ACLY inhibitors as anticancer agents is growing because ACLY overexpression increased acetyl-CoA that is necessary for lipid synthesis, and histone acetylation reactions, that regulate the expression of proteins involved in proliferation. ACLY levels are increased in CRC and other cancer types (breast cancer, hepatocellular carcinoma). ACLY inhibition brings to changes in cancer cell metabolism (Khwairakpam et al., 2015). Currently, SB-204990 has been studied in pre-clinical models of lung and prostate cancer (Hatzivassiliou et al., 2005). Indeed, ACLY inhibitors could be a potential therapeutic alternative in cancer.

#### 4.2.2 Targeting the Acetyl-CoA synthetases 2

In contrast to normal cells, cancer cells cannot obtain sufficient acetyl-CoA from pyruvate when shifted from mitochondrial respiration to aerobic glycolysis, therefore proprionate, acetate, butyrate and glutamine are other sources of acetyl-CoA in cancer cells. Acetyl-CoA is a precursor of lipid synthesis and is produced by the conversion of acetate through acetyl-CoA synthase (ACSS), which exists in three forms: ACSS-1,-2,-3. Data have shown an overexpression of ACSS-2 in different types of cancer, such as breast carcinomas, hepatocellular carcinomas (Pandey et al., 2018). In particular the role of ACSS-2 in CRC should be further studied because the fermentation by intestinal microflora is the primary source of the production of acetate. It has been shown that CRC decreased the ACSS-2 expression (Bae et al., 2017). Moreover, a correlation between breast and prostate cancer with acetate metabolism and ACSS-2 activity has been identified. In low-oxygen and lipid-depleted conditions, ACSS-2 overexpression contributes to cancer cell growth (Schug et al., 2015). Up-to-date, only one inhibitor has been described in cancer. The N-(2,3-di-2-thienyl-6-quinoxalinyl)-N’-(2-methoxyethyl)urea (CAS 508186-14-9) has been used in preclinical models of bladder cancer (Comerford et al., 2014; Wen et al., 2019). Even if the current evidence have widely proved that acetate is an essential nutrient for cancer growth, the therapeutical role of ACSS-2 is not fully explored.

### 4.3 Inhibiting fatty acid desaturation

#### 4.3.1 Targeting the stearoyl-CoA desaturase

The stearoyl-CoA desaturase (SCD) is a an enzyme with a significant role in controlling lipogenesis. SCD1 is responsible of the production of monounsaturated fatty acids (MUFAs) from saturated fatty acids (SFAs), and is a regulator of weight gain following high carbohydrate diets (ALJohani et al., 2017). The increased activity of SCD1 was observed in metastatic breast cancer, while low levels of SCD1 substrate, stearic acid in phosphatodylcholine were found in tumors associated with metastasis (Mounier et al., 2014). SCD1 overexpression reduces survival in prostate, liver, lung, kidney, and breast cancer (Ntambi, 1999). Currently, at least three different inhibitors have been identified. SSI-4 was already used in a preclinical model of hepatocellular carcinoma (Ma et al., 2017), while the betulinic acid, as well as MF-438 were both employed in preclinical models of CRC and lung cancers, respectively (Potze et al., 2016; Pisanu et al., 2017). Moreover, it has been found a positive association between CRC patient’s clinical status and SCD1 expression. In the same study, SCD1 expression was associated with cancer stem cells (CSCs)-related genes (WNT and NOTCH signaling). Targeting CSCs, as a subpopulation of cells responsible of tumor resistance and recurrence has received major attention as potential therapeutic target (Choi et al., 2019). Biochemical analysis on the role of SCD1 blockade in target stem-like cell activity in CRC may give a further rationale for the development of innovative CRC therapeutic agents.

### 4.4 Interfering with pro-inflammatory process

#### 4.4.1 Targeting the cyclo-oxigenase

Arachidonic acid is transformed through cyclooxygenase (COX) in prostaglandins and thromboxane A_2_, and exists in three isoforms. Studies have shown that COX-2 isoform is overexpressed in a series of cancers in humans, such as breast, ovarian, brain, head and neck, stomach cancer, and enhancing proliferation, apoptotitc resistence, inflammation and metastasis of cancer cells (Diakos et al., 2014). COX-2 is also released by macrophage type 2, cancer cells, and cancer-associated fibroblasts to the tumor area. Interestingly, data demonstrate that in high risk patients the use of non-steroidal anti-inflammatory drugs (NSAIDs), and of COX-2 inhibitors (COXIBs) contribute in the prevention, or delay of the onset (or recurrence) of tumors, even in patients with prior removal of CRC (Vara-Messler et al., 2015; Davis et al., 2017). Different studies have evidenced the significant potential role of NSAIDs in reducing the risk of cancer, and specifically of CRC (Thun et al., 2012; Rothwell 2013; Piazuelo and Lanas, 2015; Drew et al., 2016). In addition, the main metabolite of COX-2, PGE_2_ is present in colon, and in CRC its levels are increased, either with the disease progression, or with tumor size (Stamatakis et al., 2015; Karpisheh et al., 2019). Overall, COX inhibitors represent a promising group of drugs used as chemotherapeutic agents with a great potential for both, CRC prevention and treatment. However, further clinical studies are needed to evaluate not only the undesirable side effects, but also whether COX inhibition is effective in all types of CRC dietary interventions and cancer therapies. Apart of the energy intake, the role of diet composition has received less attention. In a normocaloric diet, 30% of energy supply comes from lipid intake, from which polyunsaturated fatty acids (PUFAs) comprise around 7–10% of daily energy intake. In different types of tumors, fatty acid oxidation (FAO) is the favored process for ATP production (Kanarek et al., 2020). Some tumors activate FAO under metabolic stress (Schafer et al., 2009; Zaugg et al., 2011; Carracedo et al., 2012). In addition, FAO also influences the redox balance. In fact, it has been shown that the human glioblastoma cell line oxidizes fatty acids, and moreover the inhibition of FAO controls the NADPH level (Pike et al., 2011). The calorie restriction has been widely studied and associated with improved health, and delay of age-related pathologies (Mihaylova et al., 2014). Calorie restriction modifies ISC function by affecting Paneth cells (Harris and Thorner, 2012; Igarashi and Guarente, 2016). EPA and DHA compete with AA for COX-2, and give rise to PGE_3_ instead of PGE_2_, a very well known prostanoid with pro-inflammatory and tumor activity (Vara-Messler et al., 2015; Koundouros and Poulogiannis, 2020). The recommended dietary ratio of ω-6:3 FAs is 1:1; although, the Western diet, rich in saturated FA, cholesterol, as well as in ω-6, has an ω-6:3 FAs ratio of 15:1, with an important role in the progression of different cancers including CRC (Vara-Messler et al., 2017; Adams et al., 2019). In fact, both a high-fat diet (HFD) rich in palmitic acid, a saturated fatty acid, as well as cholesterol-rich diet, directly alter intestinal stem cells and progenitor function. It has been demonstrated that the HFD induce obesity augmenting stemness and thus, tumorigenicity of intestinal progenitors (Beyaz et al., 2016). Considering that the energy source, metabolic activity, and nutrient requirements can be different in various type of cancers, a specific diet combined with therapies could have a potential effect in improving patient conditions.

Several studies evidence the role of enzymes, and the way the pharmacological targeting can limit the metabolic substrates for lipogenesis across several tumor types. Beside the intervention on endogenous lipid, some studies are now evaluating the high degree of inter-individual variability in metabolizing FAs (including those from diet). This complex approach may partially explain, the conflicting results coming from clinical trials of human cancer when patients were supplemented with fatty acids as a coadjutant therapy.

#### 4.4.2 Cyclopentenone prostaglandins, lipoxins and resolvins in the treatment of CRC

Cyclopentenone prostaglandins (cyPGs), PGA_1_, PGA_2_, PGJ_2_, 15-Deoxy-Δ-^12,14^-Prostaglandin J_2_ (15d-PGJ_2_) and Δ^12^-PGJ_2_ are a group of prostaglandins that increase apoptosis, resolve inflammation, have anti-metastatic and anti-angiogenic properties (Lee et al., 2021). PGA_1_ is produced from linoleic acid (Whelan and Fritsche, 2013). Among cyPGs, PGJ_2,_ 15d-PGJ_2,_ and D_12_-PGJ_2_ are expressed in human colorectal cancer cell line HCA-7.15d-PGJ_2_ has proapoptotic and antitumor activity in CRC (Chen and Tseng, 2005). Upregulation of 15-PGDH gene expression through activation of activating protein-1 (AP-1) is one of the mechanisms reported for the antitumor properties of 15d-PGJ_2_ in human colon cancer cell line HCT-116 (Park and Na, 2019a; Park and Na, 2019b). To date, it has been reported that in HCA-7 cells the major route of metabolization of PGH_2_ is through glutathione (GSH) conjugation (Cox et al., 2002).

Lipoxins (LXs) and resolvins (RVs) are proresolving lipids that display anti-inflammatory and anticarcinogenic activity. 5S,12R,18R-trihydroxy-6Z,8E,10E,14Z, 16E-eicosapentaenoic acid (RVE1) reduce the inflammation in colon mucosa (Hudert et al., 2006). Lipoxin A_4_ (LXA_4_), which levels are reduced in CRC, inhibit both the proliferation, and migration of CRC cells (Liu et al., 2019). Janakiram et al., reported that the direct administration of RVs, and LXA_4_ can have a potential role in attenuating the inflammation in colon cancer by either inhibiting, or reducing the cytokines production (Janakiram et al., 2011).

## 5 Conclusion and future perspectives

Lastly, the rewiring of FA metabolism in cancer has been widely studied because proliferating cancer cells can produce energy to obtain enough ATP for new biomass synthesis. Moreover, FA metabolism has been studied as a cell intrinsic event, considering the regulation of lipid homeostasis in oncogenic pathways, and the capacity to modify the cell membrane composition and fluidity. On the other hand, the study of FAs became interesting due to the ability to influence concomitant biological events related to tumor progression, since FAs are also able to remodel the tumor microenvironment by paracrine-signaling mechanism. In terms of therapeutic strategies, both from an intrinsic cellular point of view, as well as from its paracrine functions related to the role in the tumor microenvironment, the management of the enzymes that target FA metabolism in cancer treatment, as well as dietary interventions will improve the outcome of cancer treatment. The complexity of FA metabolism and their by-products, including the interactions with the tumor microenvironment and nutrient accessibility are important issues to be faced before developing new pharmacological targets of FA pathway’s.

We focused on the role of FAs rewiring metabolism in CRC because is a lethal disease with a high mortality rate, despite the attempts to find new treatments and improve the existing ones. Currently, the most common therapeutic approach to treat metastatic CRC foresees the combination of chemotherapy with biological agents, such as monoclonal antibodies targeting EGFR or blocking angiogenesis. A major limitation in treatment is that more than half of CRC patients are not eligible because of genetic defects, such as KRAS mutations. KRAS mutated CRC are associated with enhanced proliferation that increase the fuel need for growth and spread. In particular, KRAS-CRC may activate fatty acid synthesis to get a higher supply of energy or to induce the palmitoylation of membrane receptors, and for this reason FA pathways could have a potential metabolic vulnerability. Interestingly, plasmalogens, a group of glycerophospholipids might act as a potential biomarker for CRC screening (Fernandes et al., 2020). In specific, in colon tumors plasmalogen changes are associated with an increase in phosphatidylcholine (Dueck et al., 1996). Moreover, apoptopic genes modulation (caspase-8 and 9 activation, BBcl-2 pro-apoptotic family, PPARα and γ, LOX and prostaglandins, reduction of TCF-β-catenin genes expression (survivin)) could be potential therapeutic target for novel pharmacological compounds in CRC (Liu and Ma, 2014; Calviello et al., 2007; D'Eliseo and Velotti, 2016). Oxylipins, oxidized lipids that can be produced in enzymatic reactions, catalyzed by COX, LOX, and CYP450 are also studied recently in colorectal cancer, in which lower levels of 12-keto-LTB4 and 9-HODE and 13-HODE were shown (Zhang et al., 2019; Chistyakov et al., 2022).

Nowadays, advances in the field of CRC metabolism promise for the implementation of novel combinatorial strategies that exploit FAs dependency of cancer cells. In this context, many questions should be elucidated: How does FA metabolic pathway influence the adenoma and carcinoma sequence-progression? Which is the role of FA pathway on driving stemness and the related tumor progression? How does the FA metabolism influence metastasis? How are FAs involved on metabolic plasticity? Is the resistance to the current therapy related to FA metabolism? Managing these questions from bench-to-beside will contribute to the design of personalized therapeutic strategies that will improve the outcome of CRC.
